# Endovascular brachytherapy with iodine-125 seed strand for extensive portal vein tumor thrombus in patients with hepatocellular carcinoma

**DOI:** 10.3389/fonc.2023.1201381

**Published:** 2023-07-18

**Authors:** Zhongbao Tan, Daguang Wu, Jinhe Guo, Huanjing Wang, Jian Zhang

**Affiliations:** ^1^ Department of Interventional Radiology, The Affiliated Hospital of Jiangsu University, Jiangsu University, Zhenjiang, Jiangsu, China; ^2^ Department of Oncology, Funing County People's Hospital, Yancheng, Jiangsu, China; ^3^ Department of Interventional Radiology and Vascular Surgery, Zhongda Hospital, Nanjing, Jiangsu, China

**Keywords:** hepatocellular carcinoma, extensive portal vein tumor thrombus, iodine 125 seeds strand, endovascular brachytherapy, systematic treatment

## Abstract

**Objective:**

The aim of this study is to investigate the feasibility and effectiveness of endovascular brachytherapy with iodine-125 (I-125) seed strand for the treatment of extensive portal vein tumor thrombus (PVTT) in hepatocellular carcinoma (HCC) patients.

**Methods:**

A total of 40 HCC patients complicated by extensive PVTT who received I-125 seed strand implantation from January 2015 to December 2022 in our center were analyzed retrospectively. Endpoints included technical success rate, concurrent therapies, overall survival time, and complications. Multivariate and subgroup analyses were conducted for overall survival.

**Results:**

The successful rate of operation was 100%, and there was no operation-related death. A total of 37 patients received single I-125 seed strand implantation, and three patients received double I-125 seed strand implantation. A total of 23 patients received a concurrent therapy: transarterial chemoembolization (TACE) combined with systematic treatment (n = 6), TACE alone (n = 10), and systematic treatment alone (n = 9). At a median follow-up of 3.5 (interquartile range (IQR), 2~8.5) months, the median overall survival (OS) of all patients was 92 days (95% confidence interval (CI): 77~108). In the subgroup analysis, the median OS was 128 days (95% CI: 101~155 days) in the I-125 seed strand implantation plus systematic treatment group and was longer than that (75 days (95% CI: 36~114) of the I-125 seed strand alone group (*p* = 0.037). Multivariate analysis revealed that no systematic treatment was an independent risk factor affecting the prognosis in this study. Six patients died of upper gastrointestinal bleeding: four patients in the I-125 seed strand alone group and two patients in the combination of I-125 seed strand with systematic treatment group.

**Conclusions:**

The study shows that endovascular brachytherapy with I-125 seed strand implantation is a safe and effective treatment method for extensive PVTT in HCC patients. The combination of I-125 seed strand implantation and systematic treatment can prolong the survival time.

## Introduction

Hepatocellular carcinoma (HCC) often invades the intrahepatic portal vein and extends to the major and opposite side branch of the portal vein, thus forming portal vein tumor thrombus (PVTT). The proportions of HCC patients with PVTT range from 13% to 45% in different countries (1). HCC with PVTT is associated with a poor prognosis, and the degree of PVTT correlates with the prognosis (2, 3). Radiation therapy as an alternative therapy for treating PVTT was recommended in a few guidelines (4, 5). However, external radiation therapy may cause radiation-induced liver disease, especially for patients with a cirrhotic background (6). Recently, several reports have shown that brachytherapy using portal vein irradiation stent or stenting combined with I-125 seed strand was a safe and effective treatment method for HCC with major portal vein tumor thrombus (7–9). However, the blood flow of the portal vein cannot be recovered through stenting among HCC patients with extensive PVTT (tumor thrombus in the bilateral first portal branches with complete occlusion and major portal vein invasion, with or without the superior mesenteric vein invasion). Extensive PVTT with more tumor thrombus burden is more likely to cause portal hypertension and gastrointestinal bleeding. In our center, I-125 seed strand implantation has been performed for patients with extensive PVTT who are unsuitable for portal vein irradiation stent implantation. In this study, we conducted a retrospective study to investigate the feasibility and effectiveness of I-125 seed strand for the treatment of extensive PVTT in HCC patients.

## Materials and methods

### Patients

This retrospective study included 40 HCC patients with extensive PVTT who underwent I-125 seed strand implantation from January 2015 to December 2022. The diagnosis of HCC was according to the American Association for the Study of Liver Diseases practice guidelines or the European Association for the Study of the Liver clinical practice (10, 11). PVTT was confirmed by the presence of enhancement of a liver lesion mass expanding into the portal vein in the arterial phase and a low-attenuation intraluminal filling defect in the portal phase on enhanced CT or MRI (12). Extensive PVTT refers to tumor thrombus in the bilateral first portal branches with complete occlusion and major portal vein invasion, with or without the superior mesenteric vein invasion. Inclusion criteria: a) patients were older than 18 years, b) Child–Pugh grade A or B, c) Eastern Cooperative Oncology Group performance status (ECOG PS) of 0~2, and d) HCC patients with extensive PVTT. The exclusion criteria were as follows: a) Child–Pugh grade C, b) ECOG PS 3, and c) expected life span of less than 1 month. All patients had signed an informed consent form for I-125 seed strand implantation.

### I-125 seed strand

The diameter and length of the titanium capsule were 0.8 and 4.5 mm. I-125 seed activity was 0.6 mCi with a half-life of 59.6 days. The number (n) of I-125 seeds was determined by the length of the PVTT (L mm). N = L/4.5 + 4. I-125 seeds (CIAE-6711; Chinese Atomic Energy Science Institution, Beijing, China) were enveloped in a 4 Fr angiocatheter, in which both ends were sealed by heat.

### I-125 seed strand implantation

**Figure 1:**
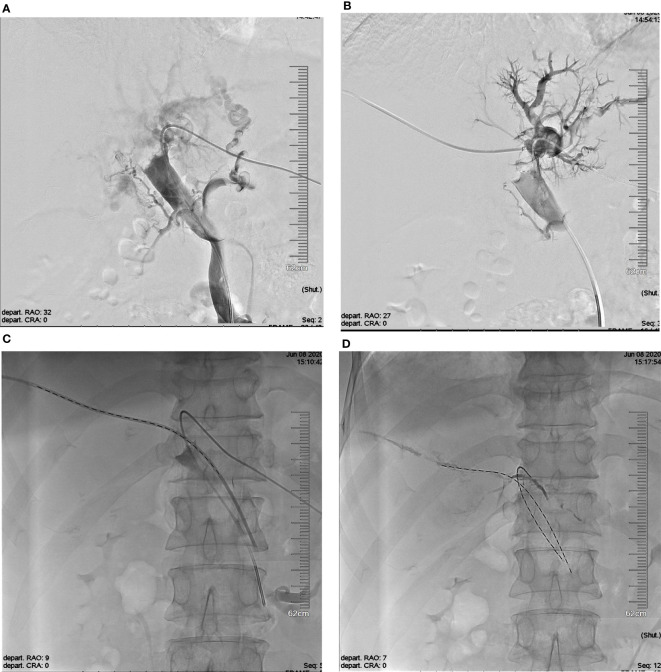
The schematic diagram of I-125 seed strand Implantation. **(A)** Photograph showing the tumor thrombus in the bilateral first portal branches with complete occlusion and major portal vein invasion. **(B)** Establishment of another I-125 seed strand implantation channel via right portal vein. **(C)** The double I-125 seed strands were delivered to the target position through the 5-Fr sheath. **(D)** The double I-125 seed strands were fixed in the PVTT. PVTT, portal vein tumor thrombus.

The operation was performed under local anesthesia by experienced interventional radiologists with more than 20 years of experience. A Chiba needle (Cook Medical, Bloomington, IN, USA) was used to puncture the second-order portal vein with color Doppler ultrasound guidance. A 6-Fr outer sleeve (Cook, Inc.) was inserted into the portal vein, followed by a 5-Fr sheath (Cordis, Miami Lakes, FL, USA). The I-125 seed strand was delivered to the target position through the 5-Fr sheath. The puncture tract then was occluded by coils or gelatin sponge (**Figure 1**).

### Follow-up

Complete blood count, liver function, alpha-fetoprotein (AFP), CT, and MRI data were collected. The primary outcome measurement was overall survival time, which was defined as the time from I-125 seed strand implantation to the date of death or last follow-up. Follow-up was censored at the occurrence of death or the end of the study period (31 May 2023). Treatments of intrahepatic parts of HCC were also collected. Complications were determined by the Common Terminology Criteria for Adverse Events 5.0 (13).

### Statistical analysis

Data were presented in mean ± standard deviation or descriptive statistics. All statistical analyses were performed using R (version 3.6.3). The chi-square test, Wilcoxon rank sum test, or Fisher’s exact test was used to compare the differences in hematological indices between preoperative and postoperative. Multivariate analysis was performed using the Cox proportional hazards model including variables with *p*-values less than 0.15 in univariate analysis. The time-to-event variables were estimated using the Kaplan–Meier method and compared by the log-rank test. *p*-Values less than 0.05 were considered statistically significant.

## Results

### Patient characteristics

According to the inclusion and exclusion criteria, a total of 40 patients with HCC complicated with extensive PVTT were included: 39 male and 1 female (**Table 1**). The average length of PVTT far from the junction of the left/right portal vein was 6.3 ± 2.9 cm. In this study, 39 cases were complicated with chronic hepatitis B virus infection. A total of 37 patients received single I-125 seed strand implantation, and three patients received double I-125 seed strands. There is no active treatment for intrahepatic parts of HCC. A total of 23 patients received a concurrent therapy: transarterial chemoembolization (TACE) combined with systematic treatment (n = 6), TACE alone (n = 10), and systematic treatment alone (n = 9). Gelatin sponge embolization of the target artery was not routinely used because of the concern that it may cause ischemic necrosis to the liver tissue. A total of 14 cases received TACE only once, and repeat TACE was performed in only two patients. A total of 15 cases received concurrent systematic therapies, including seven cases with sorafenib, three cases with Lenvatinib, and five cases with camrelizumab plus apatinib. Two patients took the target drug less than 1 month due to deterioration of liver function and gastrointestinal bleeding.

**Table 1:** Baseline characteristics of patients.

Variable	n (%)
Sex	
Male	39 (97.5%)
Female	1 (2.5%)
Age	
≤65	28 (70%)
>65	12 (30%)
Location of intrahepatic tumor	
Single liver lobe	21 (52.5%)
Whole liver lobe	19 (47.5%)
Tumor burden	
<50%	19 (47.5%)
≥50%	21 (52.5%)
Tumor type	
Infiltrative	29 (72.5%)
Nodular	11 (27.5%)
Extra-hepatic disease	
Yes	7 (17.5%)
None	33 (82.5%)
AFP	
<400 ng/ml	23 (57.5%)
≥400 ng/ml	17 (42.5%)
Child–Pugh Class	
A	19 (47.5%)
B	21 (52.5%)
ECOG PS	
0~1	23 (57.5%)
2	17 (42.5%)
Systematic treatment	
None	25 (62.5%)
Yes	15 (37.5%)
TACE	
None	24 (60%)
Yes	16 (40%)

Systematic treatment: targeted therapy or immune checkpoint inhibitor therapy.

AFP, alpha-fetoprotein; ECOG PS, Eastern Cooperative Oncology Group performance status; TACE, transarterial chemoembolization.

### I-125 seed strand implantation

**Figure 2:**
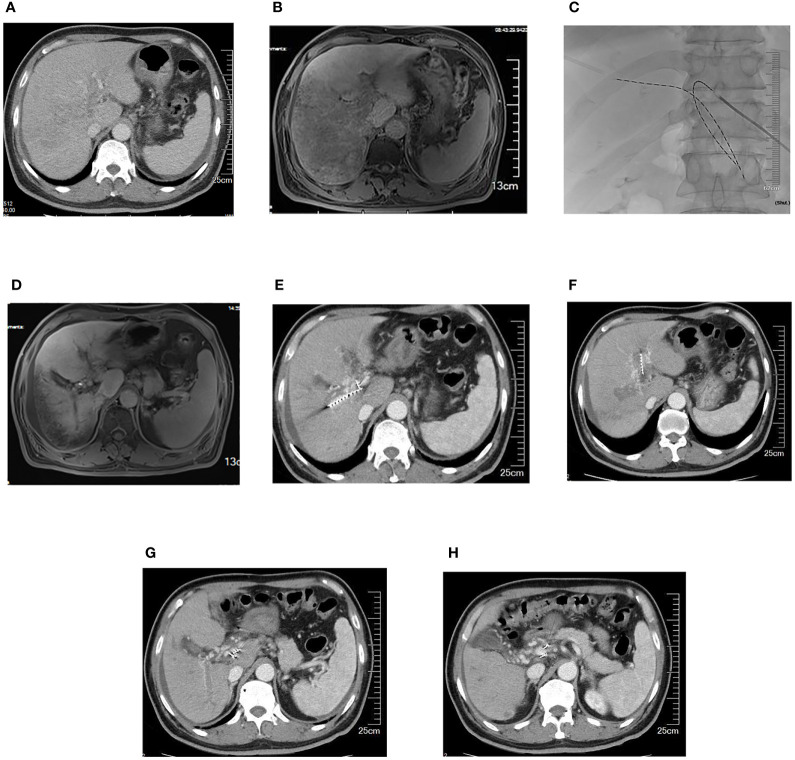
A 63-year-old male patient with HCC and extensive portal vein tumor thrombus. **(A)** Portal vein obstruction by the thrombus in bilateral portal branches and major portal vein. **(B)** Portal vein blood perfusion defect in the right liver lobe. **(C)** Two iodine-125 seed strands implanted via bilateral portal vein approach. **(D)** Portal vein blood perfusion of the right liver lobe increased. **(E-H)** Five months later, iodine-125 seed strands were still fixed in the thrombus, while the thrombus partially shrank and collateral circulation around portal vein increased. HCC, hepatocellular carcinoma.

**Figure 3:**
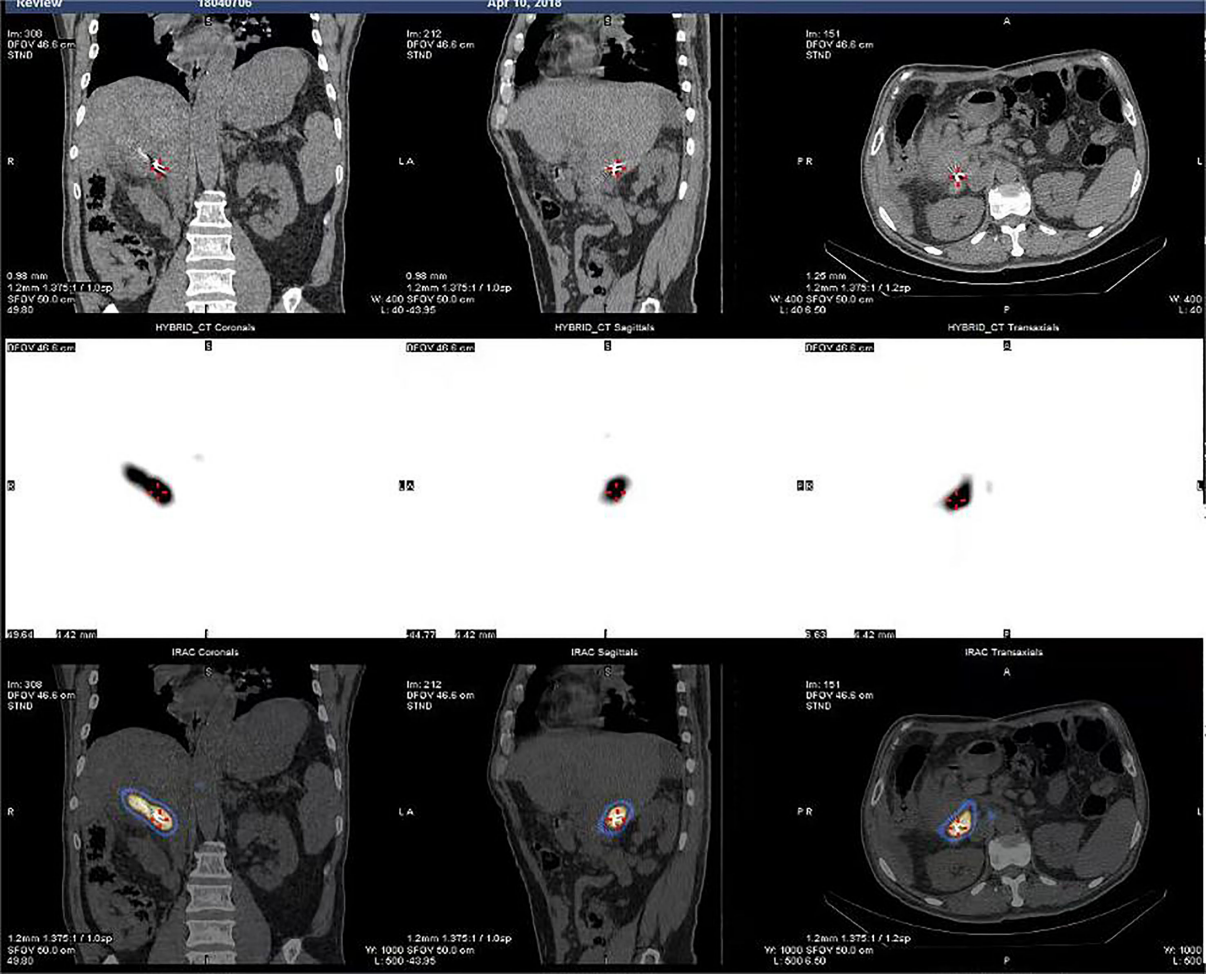
SPECTCT images showed dose distribution of I-125 seeds that surrounded the tumor thrombosis.

The success rate of the operation was 100%. A total of 43 I-125 seed strands were implanted for the treatment of 40 HCC patients with extensive PVTT. A total of 37 patients received single I-125 seed strand, and three patients received double I-125 seed strands through the bilateral portal vein approach (**Figure 2**). The average number of I-125 seeds implanted was 28 ± 10 seeds. Single-photon emission CT (SPECT)/CT images showed a distribution of I-125 seeds that surrounded the tumor thrombosis (**Figure 3**).

### Overall survival

**Figure 4:**
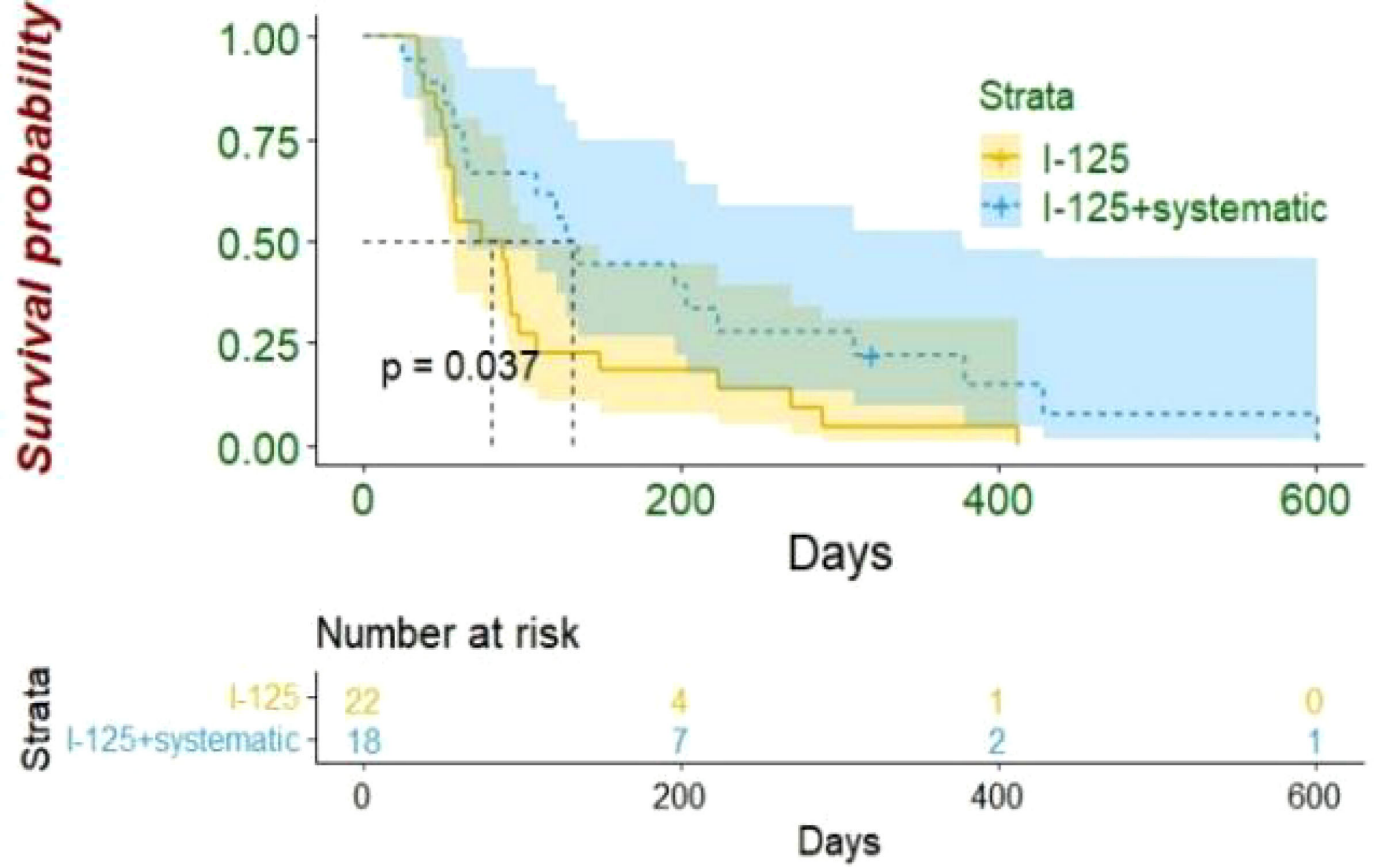
In HCC patients with extensive portal vein tumor thrombus, the median OS was better in I-125 seed strand plus systematic treatment group than I-125 seed strand alone group (128 vs. 75 days, p = 0.037). HCC, hepatocellular carcinoma; OS, overall survival.

By the end of the follow-up period (median 3.5, interquartile range (IQR), 2~8.5), 39 patients had died. The median OS of the entire patient population was 92 days in this study (95% confidence interval (CI): 77~108 days). The median OS was 128 days (95% CI: 101~155 days) for the I-125 seed strand plus systematic treatment group and 75 days (95% CI: 36~114 days) for the I-125 seed strand alone group (log-rank test, *p* = 0.037) (**Figure 4**). Of the 39 deaths during follow-up, nine patients died of gastrointestinal bleeding, and 30 patients died of liver failure. Of those nine patients who died of gastrointestinal bleeding, six patients received I-125 seed strand implantation alone, while three patients were treated with I-125 seed strand combined with systematic treatment (Fisher’s test, *p* = 1.000).

### Multi-variable analyses

The present univariate analysis results revealed that tumor burden of more than 50%, Child–Pugh grade B, and I-125 seed strand implantation alone were risk factors for poor prognosis for HCC with extensive PVTT. The multivariate Cox regression analysis demonstrated that I-125 seed strand implantation combined with systematic treatment was related to a better prognosis (**Table 2**).

**Table 2:** Univariate and multivariate analyses for patients treated with I-125 seed strand.

Variable	Univariate analysis			Multivariate analysis		
	HR	*p*	95% CI	HR	*p*	95% CI
Age						
≤65	1					
>65	1.080	0.832	0.531~2.195			
Location of tumor						
Single liver lobe	1					
Whole liver lobe	0.956	0.890	0.502~1.819			
Cirrhosis						
None	1					
Yes	1.566	0.194	0.796~3.082			
Tumor type						
Infiltrative	1					
Nodular	1.247	0.540	0.616~2.526			
Tumor burden						
<50%	1			1		
≥50%	2.037	0.034	1.056~3.928	1.935	0.101	0.879~4.259
AFP						
<400 ng/ml	1					
≥400 ng/ml	0.783	0.463	0.408~1.504			
Extra-hepatic disease						
None	1					
Yes	1.417	0.412	0.616~3.259			
Child–Pugh class						
A	1			1		
B	2.005	0.045	1.015~3.959	1.412	0.436	0.593~3.363
ECOG PS						
≤1	1					
>1	0.862	0.663	0.441~1.683			
TACE						
None	1			1		
Yes	0.564	0.115	0.276~1.150	0.631	0.249	0.289~1.379
Systematic treatment						
None	1			1		
Yes	0.495	0.040	0.253~0.969	0.410	0.014	0.201~0.839

AFP, alpha-fetoprotein; ECOG PS, Eastern Cooperative Oncology Group performance status; TACE, transarterial chemoembolization.

### Safety evaluation

No serious complications related to I-125 seed strand implantation, such as intraperitoneal hemorrhage or displacement of I-125 seeds, were recorded in this study. One patient developed a fever after I-125 seed strand implantation and improved with symptomatic treatment. No radiation hepatitis and gastroenteritis symptoms, such as decreased appetite, vomiting, and diarrhea, were observed. There were no statistically significant differences between pre- and postoperative white blood cell (WBC), platelet (PLT), hemoglobin (HB), red blood cell (RBC), total bilirubin (TB), alanine aminotransferase (ALT), and prothrombin time (PT) levels (*p* > 0.05). Aspartate aminotransferase (AST) on the fifth day after the operation was lower (*p* < 0.05). Grade 1–2 hand–foot syndrome occurred in five cases (3/15, 20%) and Grade 1 hypertension in two patients (13%) in the combination group. Gastrointestinal bleeding was observed in 12 cases: six cases (6/25) in the I-125 seed strand alone group and six cases (6/15) in combination with the systematic treatment group. Among six cases with gastrointestinal bleeding in the patient combination group, three cases presented positive stool occult blood tests and recovered normally without blocking systematic treatment.

## Discussion

This study shows that I-125 seed strand implantation is a safe and effective local treatment method for extensive PVTT in HCC patients. There was no active treatment of intrahepatic parts of HCC. The median OS of all patients was 92 days (95% CI: 77~108). The combination of systematic treatment and I-125 seed strand implantation can prolong the survival time when compared with I-125 seed strand implantation alone at 128 days (95% CI: 101~155 days) and 75 days (95% CI: 36~114 days), respectively (*p* = 0.037). Multivariate analysis demonstrated that I-125 seed strand implantation combined with systematic treatment was related to a better prognosis.

A retrospective study based on 484 HCC with different types of PVTT demonstrated that the extent of PVTT was closely related to prognosis. The median survival time of Vp1 to Vp4 was 14.6, 9.4, 5.8, and 4.8 months, respectively (3). Sorafenib alone has not been reported to significantly prolong the survival time of Vp4 PVTT with a median survival time of 3.2 months in a single-arm retrospective study by Jeong (14). Systemic treatment and a combination with locoregional therapies, such as radiation therapy, hepatic arterial infusion chemotherapy (HAIC), TACE, and Y90 transarterial radioembolization (Y90 RE), were recommended for unresectable HCC patients with PVTT, especially in Asian patients (4, 5, 15, 16). Recently, in a randomized controlled trial (RCT), Zheng et al. (17) compared sorafenib plus HAIC (n = 32) and sorafenib alone (n = 32) for advanced HCC with major PVTT (Vp3 and Vp4). The median OS was superior in the sorafenib plus HAIC group than the sorafenib alone group (16.3 *vs.* 6.5 months, *p* < 0.001). HAIC was an alternative or integrative method for HCC patients with PVTT, especially for Vp3–Vp4. In a propensity score-matching retrospective cohort study, Kim et al. (18) evaluated the efficacy of liver-directed concurrent chemoradiotherapy (LD-CCRT) (n = 52) compared with sorafenib (n = 27) in HCC patients with PVTT. After propensity score matching, the median overall survival (OS) was 4.3 and 9.8 months in the sorafenib and LD-CCRT groups, respectively (*p* = 0.002). A significant survival benefit was observed for PVTT type III and IV HCC patients in the LD-CCRT group than the sorafenib group, with 1-year of OS 41.3% *vs.* 14.3% (*p* = 0.027) and 54.5% *vs.* 0% (*p* = 0.038), respectively. In the three RCTs (SARAH, SIRveNIB, and SORAMIC) (19–21), these results showed that Y90 RE was not inferior to sorafenib for advanced HCC. Y90 RE is an effective and alternative method for advanced HCC. According to the 2021 National Comprehensive Cancer Network (NCCN) guidelines, TARE is more suitable for HCC patients with segmental or lobar PVTT (22).

The 75-day median OS of the I-125 seed strand alone group in this study was comparable to 2.7~4 months of natural median OS in HCC patients with PVTT (7, 23, 24). The survival outcomes in the combination of systematic treatment and I-125 seed strand implantation group were also worse than those in the Zheng et al. study (17) and SARAH, SIRveNIB, and SORAMIC trials (19–21). There could be several reasons for this. In this study, there was no active treatment for intrahepatic parts of HCC. The majority of the patients in this study did not receive locoregional treatments for intrahepatic tumors after iodine-125 strand implantation, such as TACE (60%), HAIC (0%), and Y90 RE (0%), and systemic treatment (62.5%). Standard TACE with lipiodol plus gelatin sponge embolization was not routinely used, which led to a less therapeutic effect for intrahepatic HCC. Repeat TACE procedures were few. Moreover, compared to the patients in those studies, the majority of the patients included in our study were relatively more late-staged with tumor burden exceeding 50% of the liver volume (52.5%). Moreover, we included patients who were not eligible for irradiation stent placement (25), and all included patients in this study with bilateral first portal branches had complete occlusion and major portal vein invasion. The more tumor thrombus burden and worse insufficient portal vein blood flow perfusion may lead to a bad prognosis (2).

Reports on the treatment of PVTT with endovascular brachytherapy using I-125 seeds are increasing gradually (26, 27). As the irregular morphology of PVTT, deep location, and the surrounding important tissues and organs are dense, improper radiation dose would lead to adjacent organ damage. Compared with external radiation therapy, brachytherapy using I-125 seed implantation has the highest local dose and more conformal dose distribution for PVTT. Hu et al. (27) conducted a retrospective study to evaluate the safety and efficacy of TACE combined with CT-guided iodine-125 implantation in HCC with the first branch of PVTT. The results demonstrated that I-125 seed implantation combined with TACE prolonged significantly the survival time compared to TACE alone (11.3 *vs.* 6 months, *p* < 0.01) and increased the local control rate of PVTT (78% *vs.* 18%, *p* < 0.01). Wang et al. (28) reported that I-125 seed strand combined with TACE can be a safe and feasible treatment option for HCC with Vp4. Compared with TACE monotherapy, the combined therapy can significantly improve the median survival (9.8 *vs.* 5.2 months, *p* = 0.024). Our center has reported that portal vein irradiation stent was a safe and effective method for the treatment of HCC with major portal vein tumor thrombus (8, 9, 25). However, stents cannot be implanted effectively into the portal vein for HCC patients with extensive PVTT in this study. Therefore, I-125 seed strand was implanted in HCC patients with extensive PVTT who were unsuitable for portal vein irradiation stent in our center. The advantages of I-125 seed strand are as follows: 1) good conformability with tumor thrombus can lead to an even and complete radioactive dose coverage, 2) I-125 seed strand is not easy to displace after implantation due to the portal vein fully filled by tumor thrombus, 3) I-125 seeds can inhibit the growth of tumor thrombus and even recover partially blood flow of intrahepatic portal vein, and 4) I-125 seed strand can prevent and delay the time of the established collateral circulation around the portal vein blocked again by the tumor thrombus, which is important for the safety of TACE or HAIC. Intrahepatic portal vein perfusion cannot be improved immediately after I-125 seed strand implantation. TACE treatment is considered only for HCC with extensive PVTT with adequate collateral circulation around the occluded portal vein and good liver function in this study. Both univariate and multivariate analyses revealed that systematic treatment was an independent risk factor affecting the prognosis of this group of patients. A study has shown that targeted therapy drugs and immune checkpoint inhibitors may have synergistic sensitization effects with I-125 seeds (29). However, excitably, although this study has poor baseline characteristics, compared with I-125 seed strand implantation alone, I-125 seed strand plus systematic treatment showed a better OS in this study. No serious complications were observed in this study. I-125 seed strand combined with systematic treatment did not increase the risk of death from gastrointestinal bleeding.

There were several limitations to this study. First, the sample size is small, and it is a single-arm retrospective study. Due to the fact that the majority of the patients in this study received single I-125 seed strand implantation, further research is needed to determine whether double I-125 seed strands are more effective. Second, as the last stage of PVTT and more tumor thrombus burden, it is difficult to fully and accurately compare this study with previous studies. A triple combination of HAIC and I-125 seed strand and systematic treatment may be a better choice for HCC patients with extensive PVTT. Third, the evaluation of tumor thrombus response was abandoned given the lack of well-recognized criteria for measuring the portal vein tumor on CT or MRI images. A larger sample of prospective randomized controlled studies should be carried out to confirm the safety and effectiveness of I-125 seed strand implantation in HCC patients with extensive PVTT.

## Conclusions

The study shows that endovascular brachytherapy with I-125 seed strand implantation is a safe and effective treatment method for extensive PVTT in HCC patients. As compared to I-125 seed strand alone, the combination of systematic treatment and I-125 seed strand implantation can prolong the survival time in HCC patients with extensive PVTT.

## Data availability statement

The original contributions presented in the study are included in the article/supplementary material. Further inquiries can be directed to the corresponding author.

## Ethics statement

This study was reviewed and approved by the ethic committee of the Affiliated Hospital of Jiangsu University (KY2023K0604). Written informed consent was waived due to its retrospective nature. All methods were carried out in accordance with declaration of Helsinki.

## Author contributions

ZT and DW: methodology, software, investigation, formal analysis, and writing–original draft. JG: technological guidance. HW: data curation and review. JZ: resources and supervision. All authors contributed to the article and approved the submitted version.
